# Comparison of Phytochemical Differences of the Pulp of Different Peach [*Prunus persica* (L.) Batsch] Cultivars with Alpha-Glucosidase Inhibitory Activity Variations in China Using UPLC-Q-TOF/MS

**DOI:** 10.3390/molecules24101968

**Published:** 2019-05-22

**Authors:** Xianan Zhang, Mingshen Su, Jihong Du, Huijuan Zhou, Xiongwei Li, Xin Li, Zhengwen Ye

**Affiliations:** 1 Forestry and Fruit Research Institute, Shanghai Academy of Agricultural Sciences, Shanghai, 201403, China; z.xn2009@163.com (X.Z.); sumingshen@saas.sh.cn (M.S.); jihonghb@126.com (J.D.); zhouhuijuanzc@163.com (H.Z.); lixiongweisea@163.com (X.L.); 2 Shanghai Key Laboratory of Protected Horticultural Technology, Shanghai, 201403, China; 3 Instrumental Analysis Center, Shanghai Jiao Tong University, Shanghai, 200240, China; qingning@sjtu.edu.cn

**Keywords:** *Prunus persica* pulp, phytochemicals, alpha-glucosidase inhibitory activity, UPLC-Q-TOF/MS, metabolomes, chemometrics, marker compounds, fruit quality

## Abstract

In order to fully understand the variation of the fruit alpha-glucosidase inhibitory activity-related phytochemical basis in the Chinese peach [*Prunus persica* (L.) Batsch], mature fruit from 33 cultivars was used for the investigation of fruit phenolic phytochemical attributes, including total phenolics, flavonoids, anthocyanins, and procyanidins, as well as the alpha-glucosidase inhibitory activity in vitro. Alpha-glucosidase inhibitory activity varied significantly among tested peach cultivars and was strongly correlated with total phenolics, total procyanidins, and total flavonoids. Untargeted UPLC-Q-TOF/MS-based metabolomics were used to comprehensively discriminate between peaches with different inhibitory activity on alpha-glucosidase. Principal component analysis (PCA) and orthogonal partial least squares discrimination analysis (OPLS-DA) were used for this process. Twenty-three differential compounds were identified between peach cultivars with high and low alpha-glucosidase inhibitory activity, and nine, including procyanidin C1, procyanidin trimer isomer 1, procyanidin trimer isomer 2, procyanidin B1, procyanidin dimer, epicatechin-epicatechin-epicatechin, phloridzin, kaempferol 3-(2’’,6’’-di-(E)-p-coumarylglucoside), and luteolin 3’-methyl ether 7-malonylglucoside, were identified as marker compounds responsible for the discrimination. Overall, variations in metabolites in peach pulp reflect the diversity in peach germplasm, and these nine compounds are good candidate markers for future genetic breeding of peach fruit with high alpha-glucosidase inhibitory activity.

## 1. Introduction

Diabetes is defined as a chronic, multifactorial disorder characterized by the failure of the body to produce insulin (type 1 diabetes) or by defects in insulin action (type 2 diabetes) [1]. The incidence of diabetes and its complications have increased significantly in the past few decades, and 422 million people have diabetes worldwide according to the World Health Organization [2]. Type 2 diabetes accounts for more than 90% of adult diabetic cases. The number of Chinese with diabetes increased to an estimated 110 million cases in 2016 [3]. Therefore, prevention and control programs are urgently needed to inhibit the rising incidence of diabetes and its complications.

Epidemiological studies have shown that a diet rich in fruits is associated with a lower risk of pre-diabetes, diabetes, and its complications [4,5]. The health benefits of fruits are partially attributed to the high content of bioactive components, such as phenolics [6]. It is increasingly being documented that phenolic compounds can affect the digestive enzymes involved in the hydrolysis of dietary carbohydrates [7,8]. Alpha-glucosidase is one of the key enzymes responsible for the hydrolysis of dietary carbohydrates [9]. The inhibition of alpha-glucosidase by phenolic-rich fruits may offer a natural dietary approach for preventing postprandial hyperglycemia.

Peaches [*Prunus persica* (L.) Batsch] are economically and nutritionally important, and they are one of the most popular fruits consumed worldwide. The peach originated from China more than 4000 years ago, and there are more than 3000 peach cultivars in the world today [10]. As the largest producer of peach fruits in the world, China (14.3 million tons, 2017 FAO data) currently has approximately more than 1000 peach cultivars [11]. Recent research efforts suggest that the consumption of peach juice and the by-product of peach juice processing can prevent hyperglycemia in obese rats [12,13,14], suggesting the potential function of peach fruits for preventing diabetes. Recently, the alpha-glucosidase inhibitory activity of peach fruit has been reported in peach cultivars grown in Poland and Portugal [15,16]. However, no extensive investigation of the alpha-glucosidase inhibitory activity of Chinese peach cultivars has been carried out. Furthermore, the chemical composition of peach fruit which leads to the difference in the activity is still unclear.

Non-targeted metabolomics analysis based on liquid chromatography-high resolution mass spectrometry allows the simultaneous detection of a wide spectrum of metabolites and rapidly provides valuable chemical information regarding molecular candidates for differentiating between cultivars [17]. With the aid of chemometrics statistical analysis, non-targeted analyses were therefore used to assess Chinese tea quality objectively and reliably [18,19]. Similarly, the huge range of peach cultivars grown in China provides important genetic resources for investigating the differences in the comprehensive metabolic profiles and their alpha-glucosidase activity. 

In the present work, the fruits of 33 Chinese peach cultivars were collected and the pulp extracts were prepared and subjected to analysis of UPLC-Q-TOF/MS and alpha-glucosidase inhibitory activity. Our work aims to contribute toward an elucidation of metabolic differences among peach cultivars with different alpha-glucosidase activity, in order to identify potential marker compounds indicative of peach alpha-glucosidase activity and provides useful data for evaluating the nutritive value to inform future breeding strategies and the consumption of peach fruits.

## 2. Results and Discussion

### 2.1. Fruit Phenolic Phytochemical Contents and Alpha-glucosidase Inhibitory Activity Evaluation

Since significant correlations were observed between phenolic contents and various bioactivities, numerous studies have been carried out to select new genotypes rich in phenolic compounds and enhanced nutritional properties [20,21,22]. In the present study, all fruits were harvested a commercial maturity, and genotypes and major fruit quality features of the 33 peach cultivars are shown in Table S1. Fruit phenolic phytochemical contents, as shown in Table 1, including the total phenolics content, total flavonoid content, total anthocyanins content, and total procyanidins content, varied significantly among the 33 cultivars tested. The white-flesh peach cultivar ‘Yu Bai’ showed the highest total phenolic content (2386.38 ± 18.44 μg gallic acid equivalent (GAE)/g fresh weight (FW)), while the yellow-flesh nectarine cultivar ‘Shuang Xi Hong’ showed the lowest value (63.61 ± 5.15 μg GAE/g FW). In addition, because phenolic compounds are, in most cases, the most abundant antioxidants in plants, the evaluation of total phenolic content gives us an approximation of the antioxidant capacity of the pulp extracts [23,24,25,26]. The total flavonoids content varied from 4.35 ± 0.27 μg rutin equivalent (RE)/g FW (‘Jin Xiang’) to 35.51 ± 3.76 μg RE/g FW (‘Mao Tao’). The total anthocyanins content of the 33 peach cultivars ranged from 1.51 ± 0.37 μg cyanidin 3-glucoside equivalent (C3GE)/g FW (‘Shuang Xi Hong’) to 138.16 ± 5.05 μg C3GE/g FW (‘Tianjin Shui Mi’). A high anthocyanins content was observed for red-flesh cultivars such as ‘Tianjin Shui Mi’ and ‘Wuhan Da Hong Pao’. Procyanidins in peach fruit have attracted much attention due to their enhanced antioxidant activity [27,28]. In this study, the total procyanidins content in the peach pulp of different cultivars varied between 0.43 ± 0.15 and 2643.68 ± 23.17 μg procyanidin B2 equivalent (PBE)/g FW, and ‘Yu Bai’ showed the highest total procyanidins content, followed by ‘Tianjin Shuimi’ (1931.5 ± 73.41 μg PBE/g FW) and ‘Feng Bai’ (1869.18 ± 38.6 μg PBE/g FW), while ‘Shuang Xi Hong’ showed the lowest total procyanidins content. All tested phenolic phytochemical contents in peach pulp showed significant germplasm diversity.

Alpha-glucosidase is a key enzyme responsible for the breakdown of oligosaccharides and disaccharides into monosaccharides suitable for absorption. The inhibition of alpha-glucosidase is one of the main strategies to counteract metabolic alterations related to hyperglycemia and type 2 diabetes [29]. Previous studies have shown that the peach fruit extract from Poland and Portugal exhibited an inhibitory effect on alpha-glucosidase, and was strongly determined by the cultivar [15,16]. However, the inhibitory effects on alpha-glucosidase of peach cultivars in China have never been investigated. As shown in Table 1, significant differences (*p* < 0.05) were found among the tested peach cultivars in the alpha-glucosidase inhibitory activity, which is consistent with previous studies [15,16], and this provides important resources for investigating the differential metabolites among cultivars with a varied inhibitory effect on alpha-glucosidase. There was a wide range of alpha-glucosidase inhibition activity, and the 50% inhibitory concentration (IC_50_) values varied from 6.92 ± 0.15 mg FW/mL (‘Yu Bai’) to 42.28 ± 3.81 mg FW/mL (‘Shuang Xi Hong’). Their distribution pattern is presented in Figure S1. The IC_50_ values of alpha-glucosidase inhibitory activity of different peach cultivars were roughly distributed normally (Shapiro-Wilk normality test, W-statistic = 0.953, *p* = 0.159).

The inhibitory effects of dietary phenolics on alpha-glucosidases have attracted great interest among researchers [8,30]. In this study, the results of the correlation analysis showed that the alpha-glucose inhibitory activity was strongly correlated with the total phenolic content (*r* = –0.7716, *p* < 0.05), total procyanidin content (*r* = –0.7829, *p* < 0.05), and total flavonoids content (*r* = –0.4313, *p* < 0.05), which suggested that phenolic compounds in peach fruit may be the main constituents responsible for the inhibitory effect on alpha-glucosidase. 

### 2.2. Relation between Metabolite Profiling and Alpha-glucosidase Inhibitory Activity

**Figure 1:**
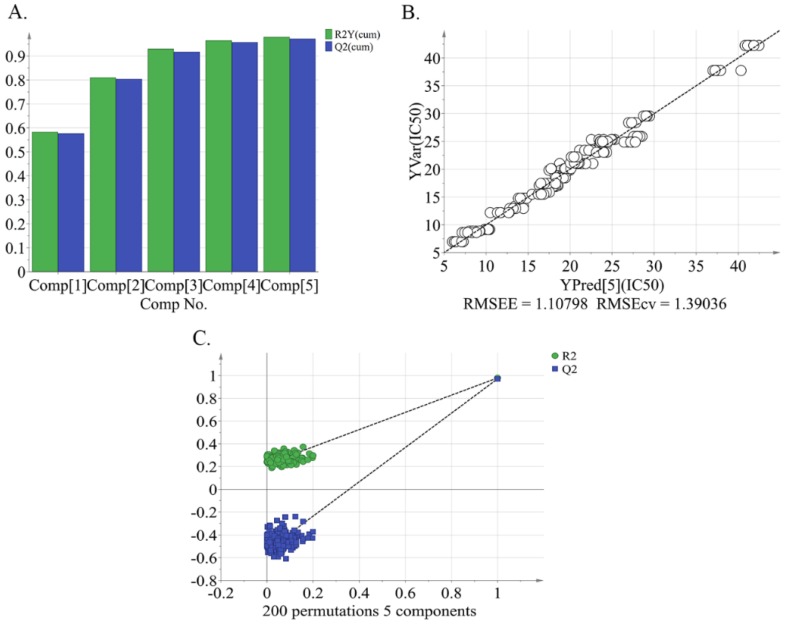
Relation of 50% inhibiting concentration (IC_50_) of peach pulp against alpha-glucosidase activity by a PLS model based on the pulp extract of 33 peach cultivars. (**A**) Summary of Fit Plot PLS; (**B**) observed vs predicted; (**C**) permutation plot for PLS [R2 = (0.0, 0.22); Q2 = (0.0, –0.531)].

A total of 1575 metabolite ions were extracted from peach pulp extracts of all 33 peach cultivars. A partial least-squares analysis (PLS) regression model was constructed to examine the relation between metabolite profiling and IC_50_ values of alpha-glucosidase inhibitory activity of peach pulp (Figure 1). The IC_50_ values of alpha-glucosidase inhibitory activity (as the dependent variable) were regressed against peak intensity from the metabolomics profile (as the independent variable) from the training sample set by means of PLS regression. The resulting values of R2Y(cum) and Q2(cum) of 0.98 and 0.922, respectively, give an indication of the good fitness and predictability of the PLS regression model (Figure 1A). Figure 1B shows the observed and predicted IC_50_ values of all tested peach cultivars. A permutation test (*n* = 200) was conducted to validate the model. The PLS regression model was found to be stable and reproducible [i.e., R2 = (0.0, 0.22), Q2 = (0.0, –0.531)] (Figure 1C). 

Phytochemical constituents contributing to the alpha-glucosidase inhibitory activity of peach pulp were obtained by filtering the variables important in the projection (VIP) > 1. A total of 545 metabolite ions from the PLS regression model were screened, and 17 metabolites, including proanthocyanidins, catechins, flavanones, dihydrochalcones, quinic acids and derivatives, were tentatively identified, based on the authentic standards, retention time (RT), accurate mass (AM), MS/MS fragment pattern, and metabolomics databases (Table 2). Correlation analyses were performed to investigate the relationship between the IC_50_ values of alpha-glucosidase inhibitory activity and the phytochemical constituents profile in different peach samples (Table 2). All of the identified 17 phytochemical constituents showed a significant correlation with the alpha-glucosidase inhibitory activity in the pulp of different peach cultivars (*p* < 0.05). Specifically, proanthocyanidins, including procyanidin C1, procyanidin B1, epicatechin-epicatechin-epicatechin, procyanidin dimer, procyanidin trimer isomer 1, procyanidin trimer isomer 2, procyanidin B2, and prunus inhibitor b, showed higher correlations (ranged from –0.615 to –0.721, *p* < 0.05), compared with catechins, flavanones, and quinic acids and derivatives (ranged from –0.355 to –0.583, *p* < 0.05) (Table 2). 

It was observed that not only the total phenolics content but also the type of phenolic compounds, played a very important role in the alpha-glucosidase inhibitory activity of peach fruit. 

Moreover, the inhibitory effect of the peach fruit can be at least partially ascribed to the 17 kinds of phenolic compounds, including chlorogenic acid, neochlorogenic acid, caffeoylquinic acid, 3-*O*-feruloylquinic acid, catechin, procyanidin B1, procyanidin B2, procyanidin dimer, procyanidin C1, procyanidin trimer isomer 1, procyanidin trimer isomer 2, epicatechin-epicatechin-epicatechin, 8,8’-methylenebiscatechin, prunus inhibitor b, prunin, naringenin, and phloridzin, which were screened based on VIP >1 by the PLS regression model. Such results provide information on the chemical compound basis of alpha-glucosidase inhibitory activity of peach fruit.

### 2.3. Metabolic Phenotype Differences between the High and Low Alpha-glucosidase Inhibitory Activity Groups

To better screen the characteristic compounds in peach pulp extracts differentiating their alpha-glucosidase inhibitory activity, the whole cultivars were artificially separated into three groups, high (< 15, eight cultivars), medium (between 10 and 25, 19 cultivars), and low (≥ 25, six cultivars), according to the IC_50_ values, hereafter referred to as groups A, B, and C, respectively (Table S2).

**Figure 2:**
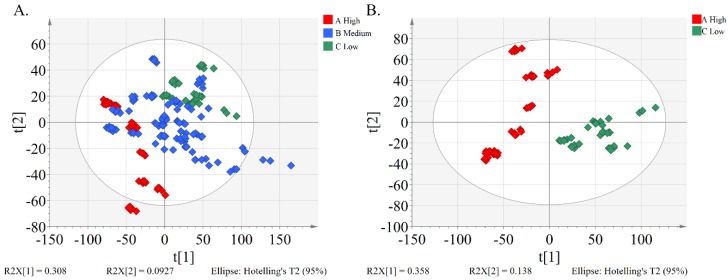
Multivariate statistical analysis of peach samples based on an unsupervised PCA model. (**A**) Unsupervised PCA plot of non-targeted metabolites analyzed by UPLC-Q-TOF/MS of 33 cultivars of peach pulp extract; (**B**) unsupervised PCA plot of two groups A (IC_50_ < 15, High) and C (IC_50_ ≥ 25, Low), which were clearly separated. Information on sample ID and IC_50_ range of groups is given in Table S2.

An overview of the differences in the metabolites of peach pulp samples was obtained using unsupervised principal component analysis (PCA) analysis, which takes into account all variables (Figure 2A). The first and second principal components explained 30.8% and 9.3% of the total variance, respectively. Quality control (QC) samples were tightly clustered in the PCA score plot, indicating the stability of the analytical platform (Figure S2). In the unsupervised PCA plot, we observe a separation of peach cultivars between group A and C, while there was not a clear separation of peach cultivars between group A and B, as well as between group B and C. The results may be due to the fact that alpha-glucosidase inhibitory activity is a continuous variable. Clear separation of group A and C along the PC1 axis was revealed by unsupervised PCA analysis performed once again for the two groups (Figure 2B). 

**Figure 3:**
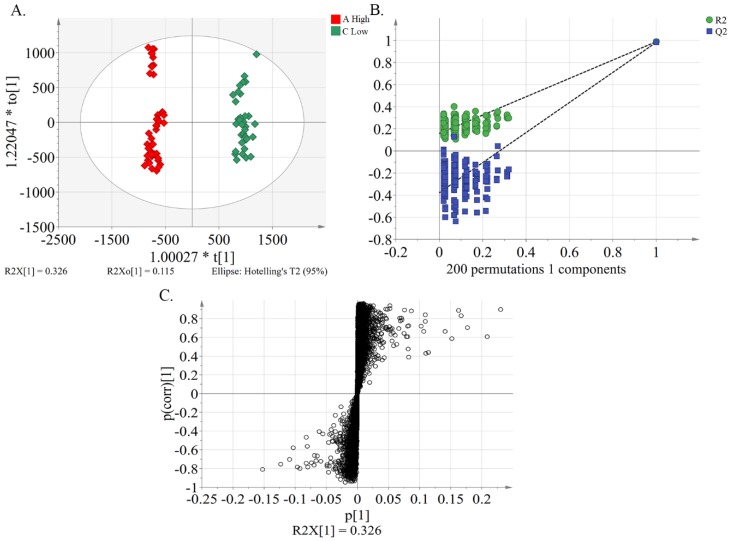
Multivariate statistical analysis of peach samples based on an OPLS-DA model. (**A**) OPLS-DA score plot showing the discrimination of the metabolome of peach cultivars between group A (IC_50_ < 15) and group C (IC_50_ ≥ 25). [R2Y(cum) = 0.988; Q2(cum) = 0.982]. (**B**) A presentation of chance permutation at 200 times used for the discrimination between group A and group C. [R2 = (0.0, 0.158); Q2 = (0.0, –0.378)]. (**C**) S-plot of OPLS-DA shows the differential metabolite expression levels of peach pulp samples between group A and group C.

Orthogonal partial least-squares discrimination analysis (OPLS-DA) is a supervised model, which filters out orthogonal metabolite variables that were not related to categorical variables, and it was used to differentiate the metabolic profile of the general differences between groups and to find the metabolites differences between groups [31]. Using the OPLS-DA model (Figure 3A), a clear discrimination was achieved between group A and C. This result suggests differences in the metabolic phenotype between the two groups. The resulting values of R2Y(cum) and Q2(cum) of 0.988 and 0.982, respectively, give an indication of the good fitness and predictability of the OPLS-DA model. A permutation test was performed 200 times to generate different random Q2 values, which were used to further test model reliability [i.e., R2 = (0.0, 0.158), Q2 = (0.0, –0.378)] (Figure 3B). Potential characteristic compounds for separation of the different inhibitory effects of peach pulp on alpha-glucosidase were obtained by filtering with the variables important in the projection (VIP) > 1, fold change (FC) > 2, and *p* < 0.01 in the statistical analysis following the construction of S-plots which present covariance (*p*) against correlation (*pcorr*) (Figure 3C).

### 2.4. Identification of Characteristic Compounds and Marker Compounds in Peach Pulp Extract Differentiating the High and Low Alpha-glucosidase Inhibitory Activity

In total, twenty-three characteristic compounds, including proanthocyanidins, catechins, flavonols, flavone, flavanones, isoflavone, dihydrochalcones, phenolic glycosides, guaianolides and derivatives, iridoid O-glycosides, and *O*-glycosyl compounds, were tentatively identified (Table 3 and Table S3). The student’s t-test also proved that these compounds were significantly different (*p* < 0.05) between group A and C. These twenty-three compounds were confirmed by comparing the RT, MS/MS fragment pattern with authentic standards, and metabolomics databases. The FCs of differential characteristic compounds between group A and C were calculated from their average peak intensity. Pearson correlation analyses between IC_50_ values of alpha-glucosidase inhibitory activity and the twenty-three differential characteristic compounds were also performed in all peach samples (Table S4). All differential characteristic components but quercetin 3-galactoside showed significant correlations with the alpha-glucosidase inhibitory activity in the pulp of different peach cultivars (*p* < 0.05).

**Figure 4:**
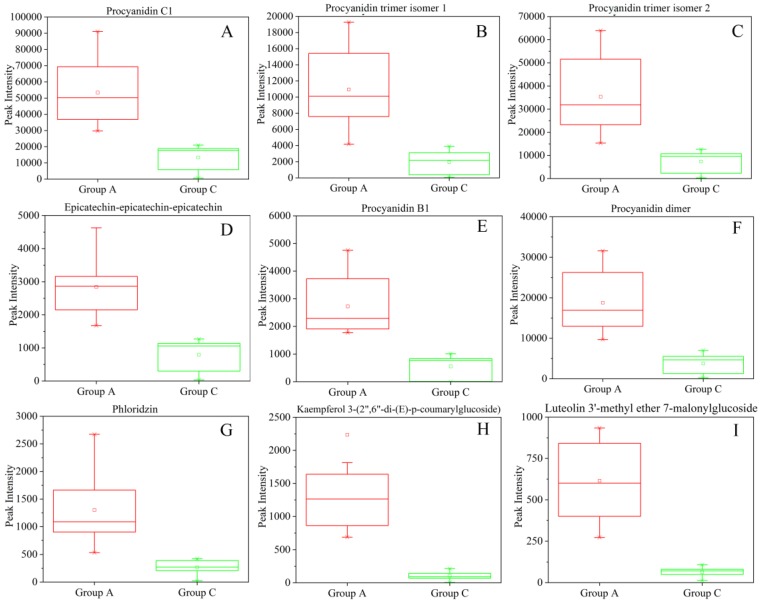
Boxplots of relative peak intensities of nine marker compounds distinguish peach cultivars with high (group A) and low (group C) alpha-glucosidase inhibitory activity. (**A**), Procyanidin C1; (**B**), Procyanidin trimer isomer 1; (**C**), Procyanidin trimer isomer 2; (**D**), Epicatechin-epicatechin-epicatechin; (**E**), Procyanidin B1; (**F**), Procyanidin dimer; (**G**), Phloridzin; (**H**), Kaempferol 3-(2’’,6’’-di-(E)-p-coumarylglucoside); (**I**), Luteolin 3’-methyl ether 7-malonylglucoside.

Receiver operating characteristic (ROC) curve analysis is widely considered to be the most objective and statistically valid method for biomarker performance evaluation [32]. ROC curves are often summarized into a single metric known as the area under the curve (AUC). AUC can be interpreted as the probability that a classifier will rank a randomly chosen positive instance higher than a randomly chosen negative one. If all positive samples are ranked before negative ones (i.e., a perfect classifier), the AUC is 1.0. An AUC of 0.5 is equivalent to randomly classifying subjects as either positive or negative (i.e., the classifier is of no practical utility), and a rough guide for assessing the utility of a biomarker based on its AUC is as follows: 0.9–1.0 = excellent; 0.8–0.9 = good; 0.7–0.8 = fair; 0.6–0.7 = poor; 0.5–0.6 = fail [33]. The plotted ROC curve for each of the confirmed differential characteristic compounds between group A and C in peach pulp under study and the calculated AUC (0.682–1.0) is shown in Table S5. Specifically, the AUC for procyanidin C1, procyanidin trimer isomer 1, procyanidin trimer isomer 2, procyanidin B1, procyanidin dimer, epicatechin-epicatechin-epicatechin, phloridzin, kaempferol 3-(2’’,6’’-di-(E)-p-coumarylglucoside), and luteolin 3’-methyl ether 7-malonylglucoside was 1.0, indicating that these nine compounds were perfect potential marker compounds for distinguishing the alpha-glucosidase inhibitory activity of peach pulp. Boxplots of peak intensities of the nine marker compounds are given in Figure 4.

In the present study, the PLS regression model was used to examine the relation between metabolite profiles and alpha-glucosidase inhibitory activity of the pulp of different peach cultivars. Phytochemical constituents contributing to alpha-glucosidase inhibitory activity were screened by filtering with VIP > 1. However, the PLS regression model cannot be used to screen for differential characteristic compounds between groups. Thus, OPLS-DA model was used to screen characteristic compounds and marker compounds differentiating the high and low alpha-glucosidase inhibitory activity of peach pulp. Differential characteristic compounds were selected on the basis of the combination of the statistically significant threshold of VIP values obtained from the OPLS-DA model and the *p*-value from a two-tailed Student’s t-test of the normalized peak area. After screening out the differential characteristic compounds, the marker compounds were further selected according to their AUC of ROC curve analysis. If the AUC is well validated, it is possible to be used as a marker compound.

So far, more than thirty phenolic compounds have been identified in the pulp of peach fruit [27]. Hydroxycinnamates, specifically, neochlorogenic acid and chlorogenic acid, have been identified as the most abundant phenolics in peach pulps, while flavan-3-ols, flavonols, and anthocyanins vary among peach cultivars [20,34]. Many of them have been shown to inhibit alpha-glucosidase, such as chlorogenic acid [35,36], neochlorogenic acid [35], catechin [35,37], phloridzin [38], oligomeric procyanidins [39,40,41], quercetin-3-galactoside [42], and so on. However, little is known about the chemical compounds basis for the difference in activity. 

In this study, the pulp extracts of all tested peach cultivars showed alpha-glucosidase inhibitory activity, which might be attributed to the combined effect of phytochemicals in peach pulps. Therefore, we emphasize the alpha-glucosidase inhibitory activity of peach fruit as a whole rather than a single component or main constituents. Recently, the highest positive correlation was observed between the content of polymeric procyanidins of peach from Poland and the alpha-glucosidase inhibitory effect [15]. Another study suggested that flavonols can interact with hydroxycinnamic acids increasing the inhibition of alpha-glucosidase [43]. Consistent with previous studies, chlorogenic acid, neochlorogenic acid, catechin, oligomeric procyanidins, and phloridzin et al., were considered to contribute at least partially to the alpha-glucosidase inhibitory activity of the peach pulp based on VIP >1 by the PLS model. Differential metabolites and marker compounds between the high and low groups with different alpha-glucosidase inhibitory activity in peach pulp were obtained in the present study, and they might also be one important part of the basis of the active compounds. The selected nine potential marker compounds (AUC =1.0), i.e., procyanidin C1, procyanidin trimer isomer 1, procyanidin trimer isomer 2, procyanidin B1, procyanidin dimer, epicatechin-epicatechin-epicatechin, phloridzin, kaempferol 3-(2’’,6’’-di-(E)-p-coumaroyl- glucoside), and luteolin 3’-methyl ether 7-malonylglucoside, can be used as indicators of alpha-glucosidase activity of peach fruit and provide useful data for evaluating the nutritive value to inform future breeding strategies and the consumption of peach fruits.

## 3. Materials and Methods 

### 3.1. Fruit Materials

Peach fruits at commercial maturity were used in the present study. The fruits of 33 cultivars were harvested from the Germplasm Collections maintained by the Forestry and Fruit Research Institute of Shanghai Academy of Agricultural Sciences in southern China (latitude/longitude: 30°53´31.79´´N/ 121°23´6.45´´E) during the summer of 2017 (Table S1). The fruits were transported to the laboratory within 3 h of harvest. Thirty of the fruits were selected for their uniformity of shape and color, and absence of disease and mechanical damage, and divided into six biological repetitions randomly. The pulps were then separated, frozen in liquid nitrogen, and stored at –80 °C before extraction and analysis.

### 3.2. Chemicals and Reagents 

HPLC-grade methanol and acetonitrile were obtained from Fisher Scientific (Pittsburgh, PA, USA). The chemical standards for quercetin-3-*O*-galactoside (≥90%), catechin (≥98%), and phloridzin (≥99%) were purchased from Sigma-Aldrich (St. Louis, MO, USA). Procyanidin B1 (>95%) and procyanidin B2 (>95%) were purchased from Solarbio (Beijing, China). Procyanidin C1 (>95%) and quercetin-3-*O*-glucoside (>98%) were purchased from Shanghai Yuanye Biotechnology Co., Ltd (Shanghai, China). Folin-Ciocalteu reagent (2 mol/L), 4-dimethylaminocinnamaldehyde (DMAC), dimethyl sulfoxide (DMSO), alpha-glucosidase (EC 3.2.1.20), and 4-nitrophenyl-α-D-glucopyranoside (PNPG) were purchased from Sigma-Aldrich (St. Louis, MO, USA). The mass spectrometry LockSpray calibration solution was leucine-encephalin and it was commercially available from Waters Corporation (ESI^−^: 554.2615, ESI^+^: 556.2771, Waters, UK). Double-distilled water (ddH_2_O) was used in all experiments and samples for UPLC-Q-TOF/MS were filtered through a 0.22 μm membrane before injection. All the other reagents were of analytical grade bought from Sinopharm Chemical Reagent Co., Ltd. (Shanghai, China).

### 3.3. Preparation of Peach Pulp Extracts 

Peach pulp extraction was performed according to previous reports, with slight modifications [20]. One gram of frozen peach pulp was extracted with 5 mL of 80% aqueous methanol with 1% formic acid by sonication for 30 min, respectively. The ultrasonic frequency was 60 kHz and the power was 300 W. The extracts were centrifuged at 4000 rpm at 4 °C for 10 min and the residue was extracted twice, as above. The supernatants of three extractions were pooled and made up to 20 mL with 80% aqueous methanol. One milliliter extract was sampled, following by centrifugation at 13000 rpm and 4 °C for 20 min. Two volumes of 500 μL supernatant were transferred to a 1.5-mL Eppendorf tube, mixed with 500 μL water purified by a Millipore Milli-Q system (Berdford, MA, USA), filtered through a 0.22 μm PTFE filter, and subjected to different kinds of phenolics contents evaluation and metabolomics analysis, respectively. Meanwhile, one volume of 500 μL supernatant was transferred to a 1.5-mL Eppendorf tube, concentrated with Concentrator plus system (Eppendorf, Germany), and then re-dissolved in 50 μL of DMSO subjected to alpha-glucosidase inhibitory activity evaluation.

### 3.4. Total Phenolics, Total Flavonoids, Total Anthocyanins, and Total Procyanidins Contents 

The total phenolics of peach pulp of different cultivars were measured using a modified colorimetric Folin-Ciocalteu method [44]. Four milliliters of ddH_2_O and 0.5 mL of appropriately diluted fruit extracts were placed in a test tube. Folin–Ciocalteu reagent (0.5 mol/L, 0.5 mL) was added to the solution and allowed to react for 3 min. The reaction was neutralized with 1 mL of saturated sodium carbonate. Absorbance at 760 nm was measured using a spectrophotometer (Shimadzu, UV-2550) after 2 h. Gallic acid was used as the standard and data were expressed as μg GAE/g FW.

Total flavonoids of peach pulp extract were determined by differential spectrophotometry with slight modification [45]. Thirty microliter extracts were taken and placed in a 96-well plate. Peach pulp extract was diluted with 210 μL methanol or 30 μL methanol solution with 2% ZrOCl_2_∙8H_2_O and 180 μL methanol. After mixing, it was maintained at 30 °C for 1 h. Then, the differential absorptivity (∆OD) of the solution before and after complexation was recorded using a microplate reader (Synergy H1, Biotek, Winooski, VT, USA) at 420 nm. Rutin was used as the standard and data were expressed as μg RE / g FW.

Total anthocyanins were determined using a modified pH differential method [46]. Peach pulp extract was diluted with 0.2 mol/L potassium chloride buffer (pH 1) or 0.2 mol/L sodium acetate buffer (pH 4.5) at a ratio of 1:4. Absorbances at 510 nm and 700 nm were measured at both pHs after 20 min under darkness. Results were expressed as μg C3GE/g FW using a molar extinction coefficient of 29,600.

Total procyanidins of peach pulp extract were measured according to the previous method, with slight modification [47]. Appropriately diluted extracts (50 μL) were added to 250 μL of DMAC solution (hydrochloric acid and ethanol; 1:9 v/v) to initiate the reaction. Absorbance at 640 nm was recorded using a microplate reader (Synergy H1, Biotek, Winooski, VT, USA) after 15 min. Procyanidin B2 was used as the standard and data were expressed as μg PBE/ g FW.

### 3.5. Determination of Alpha-glucosidase Inhibitory Activity

Alpha-glucosidase inhibitory activity was determined based on PNPG as a substrate following the slightly modified method [42]. Alpha-glucosidase (20 μL, 0.2 U/mL in 0.01 mol/L potassium phosphate buffer containing 0.2% of bovine serum albumin) and 0.1 mol/L potassium phosphate buffer (pH 6.8, 112 μL) were mixed with a test sample (8 μL). After pre-incubating at 37 °C for 15 min, 2.5 mmol/L PNPG (50 μL) was added. The reaction was incubated at 37 °C for 15 min and stopped with 0.1 mol/L Na_2_CO_3_ (80 μL). 4-Nitrophenol absorption was measured at 405 nm. Solution with DMSO instead of the sample was used as a negative control. Solution without substrate was used as a blank. Acarbose was also assayed as a standard reference. The concentration that inhibited 50% α-glucosidase activity was calculated as the IC_50_ value. The percent inhibition of α-glucosidase was calculated as follows:

$$\mathrm{Inhibition}\;(\%)\;=\;(1\;-\;\frac{{\mathrm{OD}}_{\mathrm{test}}-{\mathrm{OD}}_{\mathrm{blank}}}{\mathrm{control}{\mathrm{OD}}_{\mathrm{test}}-\mathrm{control}{\mathrm{OD}}_{\mathrm{blank}}})\;\times \;100$$

### 3.6. Condition for Metabolomic Analysis 

The non-targeted metabolites of the peach pulp extracts were analyzed using a quadrupole time of flight mass spectrometer (Vion IMS Q TOF MS, Waters Corp., Milford, MA), which was coupled with ultra-performance liquid chromatography (ACQUITY UPLC, Waters Corp., Milford, MA) system. Chromatographic separation was achieved using an ACQUITY HSS T3 column (100 mm× 2.1 mm, i.d.,1.8 μm; Waters Corp.). The column was kept at 40 °C and the flow rate was 0.4 mL/min. The mobile solutions were water with 0.1% formic acid (A) and acetonitrile containing 0.1% formic acid (B) with the gradient: 0–3 min, 0% B; 3–3.1 min, 0%–5% B; 3.1–6 min, 5%–20% B; 6–11 min, 20%–50% B; 11–15 min, 50%–100% B; 15–17 min, 100% B. Then, initial conditions were maintained for 3 min to equilibrate the column. All the samples were kept at 4 °C during the analysis. The injection volume was set to 1 μL.

The optimal MS conditions were as follows: the scan range was set at m/z 50–1000. The source voltage was 2 kV in positive mode and −2 kV in negative mode. The source temperature was 115 ℃, desolvation temperature was 450 ℃, and desolvation gas flow was 900 L/h. The MS/MS fragmentation process was accomplished at a normalized collision energy from 20 to 45 eV. The stability of the method was tested by performing repeated injections of QC samples, which were prepared by mixing aliquots of the ‘Tianjin Shuimi’, ‘Yu Bai’, and ‘Shuang Xi Hong’ samples. Online lock mass using a solution of leucine-encephalin provided a mass accuracy within 5 ppm.

### 3.7. Data Processing, Statistical Analysis, and Metabolite Identification

Experimental data of contents of total phenolics, total flavonoids, total anthocyanins, total procyanidins, and alpha-glucosidase inhibitory activity evaluation were analyzed using SPSS statistics Version 22 (SPSS Inc., Chicago, IL, USA). Data were expressed as the mean ± standard deviation (SD). Duncan’s new multiple range test was calculated for means separations in tables and *p* < 0.05 was defined as significant. Pearson correlation coefficients were calculated between alpha-glucosidase inhibitory activity and phenolic phytochemical contents at *p* < 0.05. All graphical representations were plotted by Origin Pro 9.0 (Origin lab Corporation, Northampton, MA, USA).

Raw chromatographic data acquired from the UPLC-Q-TOF/MS analysis as *.uep files were subjected to a series of pre-processing procedures, including baseline correction, denoising, smoothing, time-window splitting, deconvolution, and peak alignment using Progenesis QI software (Waters Corp.). Meanwhile, a data matrix including information on the retention time (RT), m/z, sample information, and raw abundance (peak areas of the metabolites) was generated, imported into SIMCA 14.1 (Umetrics, Umea, Sweden) for PLS, PCA, and OPLS-DA. Metabolites deemed to play important roles in distinguishing between peach samples with varied alpha-glucosidase inhibitory activity were those with the VIP > 1.0. An additional criterion for the inclusion of metabolites was that the FC between group A and C should be greater than 2 and *p*-value < 0.01 based on a student’s t-test. An ROC curve analysis was performed with MetaboAnalyst 4.0 (https://www.metaboanalyst.ca/).

The identification of metabolites was performed based on the accurate mass and mass spectrometric fragmentation patterns guided by the Human Metabolome Database (HMDB; http://www.hmdb.ca), the metabolite and tandem MS database (METLIN; http://metlin.scripps.edu), and in-house databases based on commercial standards and theoretical MS^2^ tags.

## 4. Conclusions

In the present study, significant differences were observed in the phenolic phytochemical contents, including the total phenolic, total flavonoid, total anthocyanin, and total procyanidin contents of 33 peach cultivars grown in China, as well as in the inhibitory effects on alpha-glucosidase. A UPLC-Q-TOF/MS-based untargeted metabolomics approach proved to be a reliable way to evaluate the differences in metabolites of peach pulp and identify potential marker compounds that can distinguish peach samples with alpha-glucosidase inhibitory activity variation. Nine marker compounds, procyanidin C1, procyanidin trimer isomer 1, procyanidin trimer isomer 2, procyanidin B1, procyanidin dimer, epicatechin-epicatechin-epicatechin, phloridzin, kaempferol 3-(2’’,6’’-di-(E)-p-coumarylglucoside), and luteolin 3’-methyl ether 7-malonylglucoside, were identified and confirmed based on the multivariate analysis, student’s t-test, and ROC analysis. Overall, variations in metabolites in peach pulp extracts reflect the diversity in peach germplasm, and these nine compounds are good candidate markers for the future genetic breeding of peach fruit with high alpha-glucosidase inhibitory activity. Furthermore, peach cultivars with a high content of these marker compounds may have great potential as a natural dietary source for preventing postprandial hyperglycemia.

## Figures and Tables

**Table 1:** Total phenolics, total flavonoids, total anthocyanins, and total procyanidins contents, and alpha-glucosidase inhibitory activity in the pulp extract of 33 peach cultivars from China.

Cultivars	Total Phenolics	Total Flavonoids	Total Anthocyanins	Total Procyanidins	IC_50_^*^
Bai Hua	1034.02 ± 21.32 ^h^	5.76 ± 0.22 ^fghi^	6.19 ± 1.97 ^jk^	711.8 ± 13.62 ^i^	20.99 ± 1.06 ^efghij^
Chun Mei	660.39 ± 17.9 ^k^	10.46 ± 1.2 ^de^	13.86 ± 0.25 ^defgh^	484.33 ± 7.79 ^k^	19.8 ± 0.85 ^dfghij^
Feng Bai	1805.68 ± 51.07 ^c^	6.39 ± 0.21 ^fghi^	5.86 ± 0.91 ^jk^	1869.18 ± 38.6 ^b^	9.18 ± 0.98 ^ab^
Gui Fei	1163.96 ± 14.24 ^g^	8.25 ± 0.37 ^efgh^	15.22 ± 0.64 ^def^	1066.69 ± 35.9 ^g^	12.93 ± 0.63 ^abcd^
Qiu Yue	822.26 ± 23.09 ^i^	5.88 ± 0.75 ^fghi^	6.84 ± 1.44 ^ijk^	257.19 ± 29.63 ^o^	20.11 ± 1.25 ^efghij^
Tai Nong 2	670.26 ± 19.21 ^k^	7.52 ± 0.17 ^efghi^	21.72 ± 1.12 ^c^	161.3 ± 7.05 ^p^	25.36 ± 1.02 ^jkl^
Ye Sheng Tao	1299.55 ± 19.62 ^f^	6.89 ± 1.71 ^efg^	17.62 ±1.04 ^cd^	1223.82 ± 15.08 ^e^	12.15 ± 0.44 ^abc^
Yu Bai	2386.38 ± 18.44 ^a^	16.74 ± 2.03 ^c^	5.38 ± 0.98 ^jk^	2643.68 ± 23.17 ^a^	6.92 ± 0.15 ^a^
Cheng Xiang	414.81 ± 6.54 ^no^	5.2 ± 0.25 ^ghi^	14.95 ± 0.64 ^def^	300.87 ± 6.66 ^no^	23.04 ± 2.91 ^ghijk^
Dalian 12-28	1014.28 ± 12.03 ^h^	7.91 ± 0.46 ^efghi^	14.37 ± 1.39 ^defg^	799.33 ± 10.49 ^h^	15.86 ± 0.78 ^cdef^
Dalian 1-49	641.18 ± 17.07 ^k^	5.03 ± 0.4 ^ghi^	8.99 ± 0.48 ^hijk^	463.28 ± 12.84 ^kl^	16.85 ± 1.12 ^cdefg^
Jin Xiang	793.35 ± 11.63 ^ij^	4.35 ± 0.27 ^i^	10.49 ± 1.03 ^fghij^	716.44 ± 27.63 ^i^	21.14 ± 0.26 ^efghij^
Long 1-2-4	1055.32 ± 29.97 ^h^	13.23 ± 1.36 ^d^	16.22 ± 1.67 ^de^	715.96 ± 13.49 ^i^	18.54 ± 0.4 ^cdefghi^
Zheng Huang 3	450.35 ± 83.24 ^lm^	7.52 ± 0.46 ^efghi^	15.53 ± 2.89 ^cd^	362.74 ± 8.65 ^mn^	29.6 ± 2.03 ^l^
Hei Tao	624.44 ± 17.92 ^kl^	20.02 ± 1.36 ^b^	17.58 ± 1.93 ^cd^	707.05 ± 24.5 ^i^	20.35 ± 1.48 ^cdefgh^
Tianjin Shui Mi	1976.39 ± 63.74 ^b^	18.95 ± 1.32 ^bc^	138.16 ± 5.05 ^a^	1931.5 ± 73.41 ^b^	8.91 ± 1.02 ^ab^
Wuhan Da Hong Pao	1278.43 ± 80.13 ^f^	9.38 ± 0.22 ^ef^	86.88 ± 2.15 ^b^	305.19 ± 4.46 ^no^	24.5 ± 0.87 ^ijkl^
Mao Tao	1619.42 ± 32.38 ^d^	35.51 ± 3.76 ^a^	17.74 ± 0.97 ^cd^	1176.83 ± 37.64 ^ef^	8.64 ± 0.16 ^ab^
88-4-25	1331.91 ± 41.56 ^f^	8.03 ± 0.29 ^efghi^	18.45 ± 1.04 ^cd^	1126.83 ± 25.3 ^fg^	14.77 ± 1.13 ^bcde^
Hong Shan Hu	346.88 ± 10.73 ^o^	4.97 ± 0.49 ^ghi^	5.73 ± 1.83 ^ijk^	239.01 ± 5.36 ^o^	28.32 ± 1.78 ^kl^
Huyou 002	440.35 ± 20.01 ^no^	6.5 ± 0.24 ^fghi^	8.39 ± 1.2 ^ijk^	241.86 ± 19.3 ^o^	37.75 ± 2.14 ^m^
Zao You Tao	203.46 ± 7.59 ^p^	7.97 ± 0.55 ^efghi^	9.26 ± 0.56 ^ghijk^	25 ± 1.65 ^q^	24.95 ± 0.63 ^ijkl^
Huyou 018	650.7 ± 12.57 ^k^	10.29 ± 1.42 ^de^	7.86 ± 0.96 ^ijk^	504.84 ± 12.93 ^k^	23.39 ± 0.54 ^hijkl^
Shuang Xi Hong	63.61 ± 5.15 ^q^	7.4 ± 1.5 ^efghi^	1.51 ± 0.37 ^l^	0.43 ± 1.15 ^q^	42.28 ± 3.81 ^m^
Zhong Nong Jin Hui	201.13 ± 8.04 ^p^	5.99 ± 0.45 ^fghi^	6.23 ± 0.68 ^jk^	129.33 ± 7.13 ^p^	24.87 ± 0.69 ^ijkl^
Hu 238	1494.97 ± 41.42 ^e^	6.27 ± 0.41 ^fghi^	4.13 ± 0.86 ^k^	1355.68 ± 26.44 ^d^	13.27 ± 0.48 ^bcd^
Pan Tao Huang Hou	482.64 ± 17.23 ^mn^	6.5 ± 0.63 ^fghi^	9.58 ± 1.66 ^ghij^	298.51 ± 12.31 ^no^	20.99 ± 0.51 ^efghij^
Ying Ri Er Pan Tao	662.58 ± 9.71 ^k^	7.01 ± 0.36 ^efghi^	10.38 ± 1 ^fghij^	404.79 ± 14 ^lm^	15.5 ± 1.61 ^cde^
Dalian 4-35	722.36 ± 10.04 ^jk^	5.88 ± 0.15 ^fghi^	10.13 ± 1.17 ^fghij^	612.11 ± 11.79 ^j^	22.17 ± 0.4 ^fghijk^
Zao Huang Pan Tao	362.95 ± 8.11 ^o^	4.46 ± 0.44 ^hi^	11.67 ± 1.09 ^efghi^	256.62 ± 11.81 ^o^	30.41 ± 0.32 ^jkl^
Jin Xia You Pan	397.26 ± 24.92 ^no^	7.23 ± 0.31 ^efghi^	11.94 ± 0.35 ^efghi^	796.12 ± 19.61 ^h^	18.48 ± 1.02 ^cdefghi^
Long You Pan Tao	1469.05 ± 53.34 ^e^	13.57 ± 1.72 ^d^	18.82 ± 1.15 ^cd^	1494.45 ± 31.09 ^c^	17.4 ± 2.35 ^cdefgh^
Zhong You Pan 2	404.44 ± 48.7 ^no^	4.63 ± 0.28 ^hi^	16.17 ± 0.56 ^de^	684.65 ± 29.94 ^i^	18.78 ± 0.14 ^defghi^

Results are presented as mean ± SD (*n* = 3) on a peach pulp fresh weight (g) basis. Total phenolics were calculated as μg gallic acid equivalent (μg GAE/g FW). Total flavonoids were calculated as μg rutin equivalent (μg RE/g FW). Total anthocyanins were calculated as μg cyanidin-3-glucoside equivalent (μg C3GE/g FW). Total procyanidins were calculated as μg procyanidin B2 equivalent (μg PBE/g FW). *IC_50_, Alpha-glucosidase inhibitory activity was expressed as 50% inhibitory concentration (IC_50_) of peach pulp against alpha-glucosidase activity, and the IC_50_ values were calculated as mg fresh weight of peach pulp equivalent (mg FW/mL). Values within each column followed by different letters (expressed as superscripts) were significantly different at the level of *P* < 0.05 according to Duncan’s new multiple range test.

**Table 2:** Phytochemical constituents in peach pulp contributing to alpha-glucosidase inhibitory activity and Pearson’s correlation coefficients (r) between these constituents’ profiling and the IC_50_ of alpha-glucosidase inhibiting activity.

Component	VIP ^a^	Class	Identification Level ^b^	r ^c^
Procyanidin C1	1.73	Proanthocyanidins	(i)	–0.721 *
Procyanidin B1	1.71	Proanthocyanidins	(i)	–0.719 *
Epicatechin-epicatechin-epicatechin	1.68	Proanthocyanidins	(iii)	–0.678 *
Procyanidin dimer	1.66	Proanthocyanidins	(iii)	–0.722 *
Procyanidin trimer isomer 2	1.66	Proanthocyanidins	(iii)	–0.692 *
Procyanidin trimer isomer 1	1.61	Proanthocyanidins	(iii)	–0.695 *
8,8’-Methylenebiscatechin	1.59	Catechins	(ii)	–0.583 *
Procyanidin B2	1.53	Proanthocyanidins	(i)	–0.615 *
Prunus inhibitor b	1.47	Proanthocyanidins	(ii)	–0.633 *
Catechin	1.43	Catechins	(i)	–0.527 *
Prunin	1.39	Flavanones	(ii)	–0.355 *
Phloridzin	1.38	Dihydrochalcones	(i)	–0.618 *
Naringenin	1.35	Flavanones	(ii)	–0.365 *
Neochlorogenic acid	1.25	Quinic acids and derivatives	(i)	–0.360 *
3-*O*-Feruloylquinic acid	1.20	Quinic acids and derivatives	(iii)	–0.511 *
Caffeoylquinic acid	1.15	Quinic acids and derivatives	(iii)	–0.446 *
Chlorogenic acid	1.13	Quinic acids and derivatives	(i)	–0.465 *

^a^ VIP, Variable importance for the projection. ^b^ Level of identification: (i) identified metabolites, (ii) putatively annotated compounds, and (iii) putatively characterized compounds class. ^c^ r, Pearson’s correlation coefficients, where one asterisk represents statistical significance at *p* < 0.05.

**Table 3:** Differential characteristic components in peach pulp differentiating the high (A, IC_50_ < 15, 8 cultivars) and low (C, IC_50_ ≥ 25, 6 cultivars) alpha-glucosidase inhibitory activity.

Component	VIP ^a^	*P*-Value ^b^	FC ^c^	Class	Identification Level ^d^
Procyanidin C1	12.88	2.55E-20	4.00	Proanthocyanidins	(i)
Procyanidin trimer isomer 2	10.39	1.10E-16	4.81	Proanthocyanidins	(iii)
Catechin	9.25	4.04E-13	2.50	Catechins	(i)
Procyanidin dimer	7.84	6.07E-20	5.00	Proanthocyanidins	(iii)
Procyanidin trimer isomer 1	5.91	6.57E-18	5.62	Proanthocyanidins	(iii)
Procyanidin B2	5.82	6.25E-12	3.11	Proanthocyanidins	(i)
Cynaroside A	3.34	4.39E-07	2.13	Guaianolides	(ii)
Quercetin 3-glucoside	3.00	4.11E-02	5.89	Flavonols	(i)
Procyanidin B1	2.98	1.37E-20	4.91	Proanthocyanidins	(i)
Epicatechin-epicatechin-epicatechin	2.90	3.19E-20	3.56	Proanthocyanidins	(iii)
Aucubin	2.87	9.58E-06	4.57	Iridoid O-glycosides	(ii)
Ptelatoside B	2.80	2.20E-06	4.91	Phenolic glycosides	(ii)
(1RS,2RS)-Guaiacylglycerol 1-glucoside	2.29	8.91E-06	3.67	O-glycosyl compounds	(ii)
Kaempferol 3-(2",6"-di-(E)-p-coumarylglucoside)	2.25	2.02E-05	21.22	Flavone	(ii)
Phloridzin	1.99	6.34E-16	4.88	Dihydrochalcones	(i)
Xanthotoxol glucoside	1.93	2.37E-21	2.82	Coumarin glycosides	(ii)
Prunitrin	1.85	1.51E-06	4.70	Isoflavone	(ii)
3-O-Feruloylquinic acid	1.75	4.76E-05	3.01	Quinic acids derivatives	(ii)
Epifisetinidol (4b->8) catechin	1.54	7.27E-11	8.78	Proanthocyanidins	(ii)
Luteolin 3’-methyl ether 7-malonylglucoside	1.52	7.95E-23	9.53	Flavone	(ii)
Naringenin	1.51	7.60E-04	5.84	Flavanones	(ii)
Prunus inhibitor b	1.16	2.78E-15	3.30	Proanthocyanidins	(ii)
Quercetin 3-galactoside	1.12	1.16E-02	3.16	Flavonols	(i)

^a^ VIP, Variable importance for the projection. ^b^ *p*-value: student’s t-test. ^c^ FC, fold change (group A compared with group C). ^d^ Level of identification: (i) identified metabolites, (ii) putatively annotated compounds, and (iii) putatively characterized compounds class.

## Supplementary Materials

The following are available online: Figure S1: Group distribution of peach pulp of different cultivars according to a 50% inhibiting concentration (IC_50_) against alpha-glucosidase activity (total cultivar = 33); Figure S2: Principal component analysis score diagram for mass spectrum data of samples and quality control samples; Table S1: Genotypes and major fruit quality features of the peach cultivars used in this study; Table S2: Clustering samples into three groups of high, medium, and low alpha-glucosidase inhibiting activity; Table S3: Tentative identification information of differential characteristic compounds in peach pulp between group A (IC_50_ < 15) and group C (IC_50_ ≥ 25); Table S4: Pearson’s correlation coefficients (r) between the IC50 values of alpha-glucosidase inhibiting activity and the differential characteristic components screened based on OPLS-DA model; Table S5: AUC of ROC curves of differential characteristic compounds for group A (IC_50_ < 15) versus group C (IC_50_ ≥ 25).
