# Supplementary material for: Comparison of Phytochemical Differences of the Pulp of Different Peach [Prunus persica (L.) Batsch] Cultivars with Alpha-Glucosidase Inhibitory Activity Variations in China Using UPLC-Q-TOF/MS
Source: Molecules. 2019 May 22;24(10):1968. doi: 10.3390/molecules24101968 (PMC6571656; doi:10.3390/molecules24101968)
Supplement: Supplementary file 1 [file molecules-24-01968-s001.pdf]

molecules

MDPI

Article

Comparison of phytochemical differences of the pulp of different peach [*Prunus persica* (L.) Batsch] cultivars with alpha-glucosidase inhibitory activity variation in China using UPLC-Q-TOF/MS

Xianan Zhang[1,2], Mingshen Su[1,2], Jihong Du[1,2], Huijuan Zhou[1,2], Xiongwei Li[1,2], Xin Li[3], Zhengwen Ye[1,2*]

[1] Forestry and Fruit Research Institute, Shanghai Academy of Agricultural Sciences, Shanghai, 201403, PR China; Emails: z.xn2009@163.com (X. Z.), sumingshen@saas.sh.cn (M. S.), 417001457@qq.com (J. D.), zhouhuijuanzc@163.com (H. Z.)
[2] Shanghai Key Laboratory of Protected Horticultural Technology， Shanghai, 201403, PR China;
[3] Instrumental Analysis Center, Shanghai Jiao Tong University, Shanghai, 200240, PR China; Email: qingning@sjtu.edu.cn (X. L.)
* Correspondence: yezhengwen1300@163.com (Z. Y.); Tel.: +86 021 62208175, Fax: +86 021 37195702

Received: date; Accepted: date; Published: date

Supplementary Materials

Figure S1. Group distribution of peach pulp of different cultivars according to 50% inhibiting concentration (IC50) against alpha-glucosidase activity (total cultivar = 33). Sample grouping corresponds to a roughly normal distribution (Normality test by Shapiro-Wilk, W-statistic = 0.953, P=0.159)

Figure S2. Principal component analysis score diagram for mass spectrum data of samples and quality control samples. The X-axis represents the first principal component (PC1), and the Y-axis represents the second principal component (PC2).

Table S1. Genotypes and major fruit quality features of the peach cultivars used in this study

Table S2. Clustering samples into three groups of high, medium and low alpha-glucosidase inhibiting activity.

Table S3. Tentative identification information of differential characteristic compounds in peach pulp between group A (IC50<15) and group C (IC50⩾25).

Table S4. Pearson's correlation coefficients (r) between the IC50 values of alpha-glucosidase inhibiting activity and the differential characteristic components screened based on OPLS-DA model.

Table S5. AUC of ROC curves of differential characteristic compounds for group A (IC50<15) versus group C (IC50⩾25).

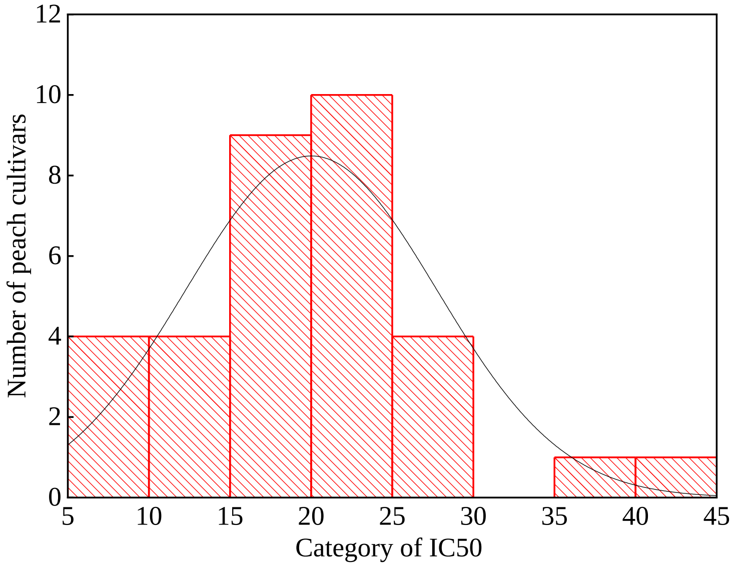

Figure S1. Group distribution of peach pulp of different cultivars according to 50% inhibiting concentration (IC50) against alpha-glucosidase activity (total cultivar = 33). Sample grouping corresponds to a roughly normal distribution (Normality test by Shapiro-Wilk, W-statistic = 0.953, P=0.159)

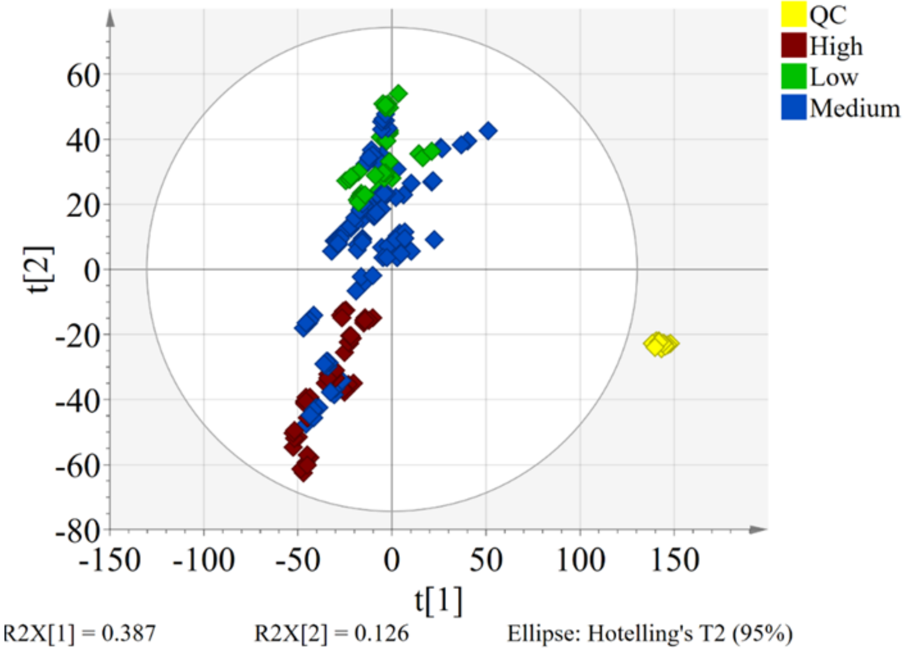

Figure S2. Principal component analysis score diagram for mass spectrum data of samples and quality control samples. The X-axis represents the first principal component (PC1), and the Y-axis represents the second principal component (PC2).

molecules

MDPI

Table S1 Genotypes and major fruit quality features of the peach cultivars used in this study.

ID[a]	Cultivar	Fruit type	Flesh color	Origin	Flesh texture	Flavor	Breeding/ Landrace	Maturity in Shanghai	Fruit weight (g)	Firmness (kg/cm^2)	TSS[b] (ºBrix)
1	Bai Hua	Peach	White	Wuxi, China	Hard melting	Strong sweet	Landrace	First ten days of August	148.5	3.9	14.8
2	Chun Mei	Peach	White	Shanghai, China	Hard melting	Sweet	Breeding	Middle of July	260.9	5.0	8.1
3	Feng Bai	Peach	White	Dalian, China	Hard melting	Sweetsour	Breeding	Middle to late July	273.5	4.0	11.3
4	Gui Fei	Peach	White	Shandong, China	Non-meltng	Sweet	Breeding	Early to late August	178.8	5.3	15.9
5	Qiu Yue	Peach	White	Shanghai, China	Hard melting	Sweet	Breeding	Middle to late August	325.6	4.4	12.9
6	Tai Nong 2	Peach	White	Taiwan, China	Soft melting	Sweet	Breeding	First ten days of July	101.1	1.5	8.4
7	Ye Sheng Tao	Peach	White	Zhejiang, China	Hard melting	Sweet	Landrace	First ten days of August	76.3	3.8	19.7
8	Yu Bai	Peach	White	Zhengzhou, China	Non-meltng	Sweet	Breeding	First ten days of July	156.0	6.8	10.8
9	Cheng Xiang	Peach	Yellow	Dalian, China	Soft melting	Soursweet	Breeding	Last ten days of June	168.4	0.5	10.2
10	Dalian 12-28	Peach	Yellow	Dalian, China	Non-meltng	Sour	Breeding	Middle of July	199.7	3.0	16.0
11	Dalian 1-49	Peach	Yellow	Dalian, China	Non-meltng	Soursweet	Breeding	Last ten days of July	167.6	3.8	9.4
12	Jin Xiang	Peach	Yellow	Shanghai, China	Hard melting	Sweet	Breeding	Middle to late July	259.7	0.8	10.7
13	Long 1-2-4	Peach	Yellow	Gansu,China	Non-meltng	Soursweet	Landrace	First ten days of August	150.6	3.3	13.4
14	Zheng Huang 3	Peach	Yellow	Zhengzhou, China	Non-meltng	Soursweet	Breeding	First ten days of July	192.6	1.6	8.4
15	Hei Tao	Peach	Red	Shandong, China	Hard melting	Sour	Landrace	Early of September	71.0	4.7	11.4
16	Tianjin Shui Mi	Peach	Red	Tianjin, China	Hard melting	Sour	Landrace	First ten days of July	142.7	0.7	8.5
17	Wuhan Da Hong Pao	Peach	Red	Wuhan, China	Hard melting	Sweet	Landrace	Last ten days of June	132.7	0.6	9.5
18	Mao Tao	Peach	Green	-	Hard melting	Sour, astringent	Wild	Last ten days of August	51.3	2.4	13.9
19	88-4-25	Nectarine	White	Beijing, China	Hard melting	Sweet	Breeding	First ten days of August	111.6	2.7	19.3
20	Hong Shan Hu	Nectarine	White	Beijing, China	Soft melting	Strong sweet	Breeding	First ten days of July	131.5	2.6	12.6
21	Huyou 002	Nectarine	White	Shanghai, China	Hard melting	Sweet	Breeding	First ten days of June	128.2	4.4	9.8

22	Zao You Tao	Nectarine	White	Shandong, China	Hard melting	Sweet	Landrace	Middle of July	72.1	6.2	12.6
23	Huyou 018	Nectarine	Yellow	Shanghai, China	Hard melting	Sweet	Breeding	Early to middle June	212.9	5.4	11.1
24	Shuang Xi Hong	Nectarine	Yellow	Zhengzhou, China	Hard melting	Sweet	Breeding	Last ten days of June	128.9	6.3	13.2
25	Zhong Nong Jin Hui	Nectarine	Yellow	Zhengzhou, China	Hard melting	Sweet	Breeding	Middle of June	150.8	4.0	9.9
26	Hu 238	Flat peach	White	Shanghai, China	Hard melting	Sweet	Breeding	Middle to late July	112.7	1.8	14.8
27	Pan Tao Huang Hou	Flat peach	White	Zhengzhou, China	Hard melting	Sweet	Breeding	Early to middle June	235.8	0.7	10.3
28	Ying Ri Er Pan Tao	Flat peach	White	Xinjiang, China	Hard melting	Strong sweet	Landrace	Middle to late July	92.4	1.2	15.4
29	Dalian 4-35	Flat peach	Yellow	Dalian, China	Soft melting	Strong sweet	Breeding	First ten days of July	165.5	0.6	10.2
30	Zao Huang Pan Tao	Flat peach	Yellow	Zhengzhou, China	Soft melting	Strong sweet	Breeding	Last ten days of June	142.2	3.2	10.8
31	Jin Xia You Pan Tao	Flat, nectarine	Yellow	Jiangsu, China	Hard melting	Sweet	Breeding	Middle to late July	163.4	5.6	13.3
32	Long You Pan Tao	Flat, nectarine	Yellow	Zhengzhou, China	Hard melting	Soursweet	Breeding	First ten days of August	123.3	5.6	20.2
33	Zhong You Pan Tao 2	Flat, nectarine	Yellow	Zhengzhou, China	Hard melting	Strong sweet	Breeding	First ten days of July	158.2	5.2	14.1

[a] Thirty-three of peach cultivars used in this study were numbered according to the fruit type and flesh color. [b] TSS, Total soluble solid content. Data were expressed as the means of ten fruits.

molecules

Table S2 Clustering samples into three groups of high, medium and low alpha-glucosidase inhibiting activity.

Group	Rank	Size	Alpha-glucosidase inhibiting activity *	Cultivar ID
A	High	8	<15	3, 4, 7, 8, 17, 19, 20, 27
B	Medium	19	≥15&<25	1, 2, 5, 9, 10, 11, 12, 13, 14, 16, 18, 23, 24, 26, 28, 29, 31, 32, 33
C	Low	6	≥25	6, 15, 21, 22, 25, 30

*alpha-glucosidase inhibiting activity were expressed as 50% inhibiting concentration of peach pulp against alpha-glucosidase activity that were calculated as mg fresh weight of fruit pulp equivalent (mg/ml).

Table S3 Tentative identification information of differential metabolites in peach pulp between group A (IC50 < 15) and group C (IC50 ≥ 25), and information of standards used in this study.

Compounds	Neutral (m/z)	Observed (m/z)	Mass Error (ppm)	RT (min)	Ion detected	Major fragments m/z (%)	Molecular formula	Ref.	Level of identification*
Aucubin	346.126	327.1081	-1.23	3.38	M-H2O-H	165.0550(100), 147.0445(52), 119.0495(15), 107.0496(32)	C15H22O9	HMDB	2
(1RS,2RS)-Guaiacylglycerol 1-glucoside	376.1364	375.1291	-1.64	3.44	M-H	272.0879(11),195.0654(41)	C16H24O10	HMDB	2
Procyanidin C1 isomer 1	866.2058	865.1977	0.90	3.61	M-H	713.1509(28), 695.1405(83), 413.0875(34), 287.0555(100)	C45H38O18	HMDB	3
Luteolin 3'-methyl ether 7-malonylglucoside	548.1166	547.1094	0.06	3.77	M-H	341.0678(100), 219.0291(8), 191.0350	C25H24O14	HMDB	2
Procyanidin B1	578.1424	577.1349	-0.40	3.96	M-H	425.0875(27), 407.0770(100), 289.0713(79)	C30H28O13	Standard	1
Prunitrin	446.1213	891.2343	-1.14	3.96	2M-H	577.1349(66), 289.0713(79), 381.0974(3.34), 245.0447(6.22)	C22H22O10	HMDB	2
Procyanidin B2	578.1424	577.1345	-1.20	4.09	M-H	425.0890(30), 407.0784(100), 289.0715(97), 245.0803(25), 125.0247(22)	C30H28O13	Standard	1
Procyanidin C1	866.2058	865.1979	-0.70	4.12	M-H	577.1345(100), 425.0874(53), 287.0555(36)	C45H38O18	Standard	1
Epicatechin-epicatechin-epicatechin	864.1902	863.1823	-0.73	4.14	M-H	739.1668(18), 577.1345(100), 413.0875(25), 287.0555(36)	C45H36O18	HMDB	3
Catechin	290.079	289.0716	-0.20	4.20	M-H	245.0812(100), 203.0706(71), 151.0393(46)	C15H14O6	Standard	1
Xanthotoxol glucoside	364.0794	727.1501	-2.74	4.29	2M-H	329.0228(27), 301.0361(5), 261.0398(32), 243.0292(100), 245.0448(67)	C17H16O9	HMDB	3

Prunus inhibitor b	560.1319	605.1304	0.68	4.57	M+FA-H	407.0772(100), 287.0556(90), 245.0448(15), 161.0236(20)	C30H24O11	HMDB	2
Procyanidin C1 isomer 2	866.2058	865.1971	-1.67	4.59	M-H	739.1662(25), 575.1194(66), 405.0614(39), 287.0556(100)	C45H38O18	HMDB	3
Procyanidin dimer	578.1424	577.1348	-0.66	4.86	M-H	407.0772(80), 289.0711(100), 161.0238(10), 125.0239(30)	C30H28O13	HMDB	3
Kaempferol 3-(2”,6”-di-€-p-coumarylglucoside)	740.1741	739.1659	-1.27	5.02	M-H	629.1312(2), 435.0732(35), 339.0515(31), 325.0355(2), 227.0350().4）	C39H32O15	HMDB	2
3-O-Feruloylquinic acid	368.1103	367.1030	-1.31	5.41	M-H	367.1030(44), 161.0449(2), 113.0239(1), 101.0238(0.4)	C17H20O9	HMDB	3
Cynaroside A	444.1993	443.1919	-0.59	5.70	M-H	443.1921(100), 399.2024(2), 303.1212(4), 281.1391(1), 263.1285(4), 241.1075(15), 221.0787(4), 205.0862(9)	C21H32O10	HMDB	2
Quercetin 3-O-galactoside	464.0953	463.0881	-0.15	5.77	M-H	301.0339 (100)	C21H20O12	Standard	1
Quercetin 3-glucoside	464.0953	463.0881	-0.19	5.88	M-H	301.0339(100), 271.0244(67), 255.0294(41)	C21H20O12	Standard	1
Epifisetinidol (4b->8) catechin	562.1475	561.1399	-0.39	6.07	M-H	391.0818(10), 301.0707(3)	C30H26O11	HMDB	2
Ptelatoside B	428.1679	427.1607	-0.67	6.75	M-H	293.0890(25), 233.0660(63), 133.0652(100)	C20H28O10	HMDB	2
Naringenin	272.0685	271.0605	-2.74	6.77	M-H	177.0186(41), 151.0030(100), 119.0497(35)	C15H12O5	Metlin	2
Phloridzin	436.1366	435.1293	-0.83	7.31	M-H	273.0763(100),167.0342(43)	C21H24O10	Standard	1
Standards									
Neochlorogenic acid	354.0951	353.0878	0.0	3.86	M-H	191.0557(100), 179.0344(51), 135.0452(36)	C16H18O9		
Chlorogenic acid	354.0951	353.0875	-0.80	4.18	M-H	191.0559(100)	C16H18O9		
Catechin	290.0790	289.0713	-1.60	4.20	M-H	245.0819(82), 203.0706(78), 205.0499(45), 151.0395(30), 123.0455(50), 125.0455(40)	C15H14O6		
Procyanidin B1	578.1424	577.1352	0.10	3.96	M-H	451.1057(20), 425.0900(40), 407.0788(100), 289.0715(90)，245.0809(18), 161.0236(10), 125.0241(25)	C30H26O12		

Procyanidin B2	578.1424	577.1340	-2.0	4.09	M-H	451.1055(18), 425.0898(50), 407.0784(100), 289.0715(90), 245.0804(10), 161.0236(10), 125.0246(20)	C30H26O12
Procyanidin C1	866.2058	865.1972	-1.5	4.12	M-H	739.1781(10), 713.1612(13), 695.1491(14), 577.1409(22), 575.1252(17), 425.0899(13), 407.0788(18), 289.0712(13), 287.0558(20)	C45H38O18
Quercetin 3-*O*-galactoside	464.0955	463.0878	-0.9	5.77	M-H	301.0363(79), 271.0253(20), 255.0301(10)	C21H20O12
Quercetin 3-*O*-glucoside	464.0955	463.0880	-0.4	5.88	M-H	301.0355(48), 271.0237(15), 255.0288(10)	C21H20O12
Phloridzin	436.1369	435.1289	-1.8	7.31	M-H	273.0765(100), 179.0339(9), 167.0344(60)	C21H24O10

*Level of identification: 1, identified metabolites on the basis of actual mass (AM), MS/MS, databases, and standards; 2, putatively annotated compounds on the basis of AM, MS/MS, and databases; 3, putatively characterized compounds class on the basis of AM and MS/MS.

Table S4. Pearson's correlation coefficients (r) between the IC50 values of alpha-glucosidase inhibiting activity and the differential characteristic components screened based on OPLS-DA model.

**Component**	**r [a]**
Procyanidin C1	-0.721 *
Procyanidin C1 isomer 2	-0.692 *
Catechin	-0.527 *
Procyanidin dimer	-0.722 *
Procyanidin C1 isomer 1	-0.695 *
Procyanidin B2	-0.615 *
Cynaroside A	-0.397 *
Quercetin 3-glucoside	-0.197 *
Procyanidin B1	-0.719 *
Epicatechin-epicatechin-epicatechin	-0.678 *
Aucubin	-0.348 *
Ptelatoside B	-0.310 *
(1RS,2RS)-Guaiacylglycerol 1-glucoside	-0.372 *
Kaempferol 3-(2",6"-di-(E)-p-coumarylglucoside)	-0.447 *
Phloridzin	-0.618 *
Xanthotoxol glucoside	-0.695 *
Prunitrin	-0.469 *
3-O-Feruloylquinic acid	-0.511 *
Epifisetinidol (4b->8) catechin	-0.645 *
Luteolin 3'-methyl ether 7-malonylglucoside	-0.707 *
Naringenin	-0.365 *
Prunus inhibitor b	-0.633 *
Quercetin 3-galactoside	-0.111

[a] r, Pearson's correlation coefficients, one asterisk represents statistical significance at $p < 0.05$.

Table S5. AUC of ROC curves of differential characteristic compounds for group A (IC50 < 15) versus group C (IC50 ≥ 25).

**Compounds**	**AUC**
Procyanidin C1	1.0
Procyanidin C1 isomer 2	1.0
Procyanidin dimer	1.0
Procyanidin C1 isomer 1	1.0
Procyanidin B1	1.0
Epicatechin-epicatechin-epicatechin	1.0
Kaempferol 3-(2'',6''-di-(E)-p-coumarylglucoside)	1.0
Phloridzin	1.0
Luteolin 3'-methyl ether 7-malonylglucoside	1.0
Epifisetinidol (4b->8) catechin	0.999
Xanthotoxol glucoside	0.998
Catechin	0.931
Procyanidin B2	0.931
Prunus inhibitor b	0.914
Prunitrin	0.894
Aucubin	0.829
(1RS,2RS)-Guaiacylglycerol 1-glucoside	0.813
Naringenin	0.813
3-O-Feruloylquinic acid	0.788
Cynaroside A	0.781
Ptelatoside B	0.771
Quercetin 3-galactoside	0.733
Quercetin 3-glucoside	0.682
